# Tait’s Operation—Recovery

**Published:** 1887-04

**Authors:** B. F. Kingsley

**Affiliations:** San Antonio, Texas


### TAIT'S OPERATION—RECOVERY.

*By B. F. Kingsley, M. D., San Antonio, Texas.*

For Daniel's Texas Medical Journal.

IN view of the interest attaching to the subject of laparotomy for various causes, allow me, in accordance with your suggestion, to report the following case.

The woman, *æt* 24, one child, a boy 5 years old; two miscarriages; considerably emaciated, chronic constipation; a history of general pelvic inflammation. Three years continuous but unavailing treatment; womb immobile.

With Dr. Cupples in consultation, the diagnosis of my sister, Josephine Kingsley, M. D., was confirmed, and her opinion that an operation was the only means of relief, was endorsed. Accordingly the operation was decided upon, and done, March 13, with the assistance of Drs. Cupples, Watts, Barnitz, Johns, Tyler, and J. Kingsley.

A three inch median incision was made, and the peritoneum opened without delay. Everywhere there were firm adhesions. The left ovary was seized, and the adhesions broken up with some difficulty, the tube ligated close to the uterus, and removed. It was much enlarged on a cyst as large as a hens egg, involving the ovary and fimbriated extremity of the tube in its origin; there were also a number of minute cysts studding ovarian stroma.

The right ovary and tube were more adherent; and rather than break up the adhesions the ovary alone was detached, and a ligature placed around the pavillion of the tube, and the ovary removed, leaving the tube in situ; first, however, a small hematoma of the broad ligament, the size of a hickory nut, was ligated and cut away. This ovary was found to contain two pus cavities, filled, one with bloody pus, the other with laudable pus. The oozing was checked, and the wound closed in the usual way. The only antiseptic precautions being a careful preparation of the sponges, and boiled water reduced to near the normal temperature.

Chloroform vomiting continued for five days; and on the 27th an abscess at lower deep suture point (which, by the way, was removed on the seventh day) had developed and broken, sending the temperature up to 104:2, which became normal the next day by liberal use of quinine, and a dose of antipyrine. I attribute the abscess to the excessive vomiting.

The patient now looks and feels better than before the operation.

The chief features of interest in this case being, the complete failure of three years of medication and five Emmet's operations, "catarrhal phthisis" or lung trouble, which was supposed to be a contra-indication to the operation; reluctance of her previous attending physicians to advise operation; the rarity and unique character of the pathology involved in the case; the result of the operation, which abundantly confirmed the diagnosis; the protracted vomiting and abscess, in spite of which she is making, up to this point, a rapid and, to her, happy recovery, and looks and feels better than before the operation.

*April 7, 1887.*

